# Resilience as a *Buffering* Variable Between the Big Five Components and Factors and Symptoms of Academic Stress at University

**DOI:** 10.3389/fpsyt.2021.600240

**Published:** 2021-07-08

**Authors:** Jesús de la Fuente, María Carmen González-Torres, Raquel Artuch-Garde, Manuel Mariano Vera-Martínez, Jose Manuel Martínez-Vicente, Francisco Javier Peralta-S'anchez

**Affiliations:** ^1^School of Education and Psychology, University of Navarra, Pamplona, Spain; ^2^School of Psychology, University of Almería, Almería, Spain; ^3^Department of Health Sciences, School of Psychology, Public University of Navarra, Pamplona, Spain; ^4^School of Psychology, University of Granada, Granada, Spain

**Keywords:** Big Five model, resilience, stress and factor symptoms, SEM model, university

## Abstract

The aim of this cross-sectional study was to establish predictive relationships of the Big Five personality factors (according to their self-regulatory level), together with resilience (proactive and reactive factors), for factors and symptoms of academic stress related to teaching and learning in the University context. A total of 405 female undergraduate students were selected, and completed questionnaires that had been previously validated in Spanish University students (Big Five personality factors, resilience, and academic stress symptoms and factors). A linear, ex-post facto design was used, including linear regression, Structural Equation Modeling (SEM), and mediational analyses. Specific linear regression showed the expected gradation: that self-regulatory personality factors (conscientiousness, extraversion) were positive linear predictors of proactive resilience, as well as significant negative predictors of stress factors and symptoms of academic stress; while the non-regulatory personality factors (openness to experience, agreeableness) showed little relationship. By contrast, the dysregulatory personality factor (neuroticism) was a negative predictor of proactive resilience, a positive predictor of reactive resilience, and positively predicted academic stress factors in the teaching and learning process, as well as stress symptoms. SEM general analysis showed that personality factors positively predicted resilience, and resilience negatively predicted factors and symptoms of academic stress. Specific mediational model analysis, with each personality factor, confirmed the different mediating relationships that appeared in the linear regression analyses. These results are discussed from the perspective of promoting resilience and healthy personalities in the University context. Implications for addressing academic stress at University are discussed.

## Introduction

Stress in the University context is a natural phenomenon (1) and has been a common problem for college students in every era (2). Nonetheless, the college environment seems to be increasingly stressful in recent decades (3, 4) and the experience of perceived stress, anxiety, and feeling overwhelmed is widespread among college students, including those who succeed, as documented by different surveys (5, 6) and diverse publications (7–11). Reddy et al. (12) claim that this has become a grave reality to the point of becoming a “career stopper.”

Students are subjected to many different sources of stress, especially *academic stressors*, which are well-documented in the literature (3, 13, 14). The evaluative processes that are involved in one's subjective experience of stress (15) are affected by contextual and psychosocial factors (8, 16–18).

The way we react to stress varies greatly between individuals, and the students need develop personal resources to successfully progress through higher education, despite its constant challenges (19). Recent years have seen growing interest in these resources what are called *non-cognitive variables*—also known as personal skills, personal qualities, character traits, psychosocial skills, and soft skills (20–23)—for their important impact on educational achievement, success in the job market, career and life success, and well-being (24, 25). Among the *non-cognitive skills* that may increase vulnerability to stress, or, constitute protective resources for coping, two important constructs have been emphasized, and will be addressed in this study: personality traits (BF model) and resilience (19). In research on stress at University, particularly from the standpoint of health sciences, certain personality traits are considered critical non-cognitive variables that make up a resilient personality, able to manage stress successfully (19). However, from the *Vulnerability- stress model* or diathesis-stress model (26), individuals may possess a pre-disposition toward certain disorders, as is the case with neuroticism, where individuals have the tendency to feel overwhelmed by stress. On the other hand, certain qualities linked to resilience can be cultivated; these qualities protect against stress and strengthen one's resistance (27).

The current study seeks to establish whether the BF traits and Resilience may be significant predictors of students' stress factors and symptoms. Another important objective is to examine the role of Resilience in mediating between the BF traits and stress factors and responses. To date, there has been little analysis of this role in the University context (28–30).

### Academic Stress in the University Context: Teaching-Learning Factors and Symptoms

Stress is a complex phenomenon and many theoretical models have been proposed to explain its etiology. “The transactional theory of stress and coping, developed by Lazarus and Folkman, has been particularly instrumental in shaping stress and coping research over the past five decades” [(31), p. 351]. According to their model (15), individuals are constantly evaluating the stimuli in their environment, and this evaluation generates emotions. Stimuli that are considered threatening, challenging or harmful result in distress, and coping strategies are then activated to manage emotions or to directly address the stressor itself. From this perspective, stress is defined as exposure to stimuli that are appraised as harmful, threatening, or challenging, exceeding the individual's capacity to cope [(31); p. 352].

The study of academic stress at University, as a factor that is detrimental to psychological health or emotional well-being, is a highly current research topic (10, 32, 33). In the University context, numerous potential factors of stress have been documented and categorized, such as academics, the learning environment, campus culture, interpersonal and personal issues (9, 34).

#### Academic Stress Factors From the Teaching and Learning Process

Academic stress factors themselves can be classified into three general groups: (1) those related to performance assessment; (2) those related to a heavy workload, and (3) other conditions of the teaching-learning process, such as social relationships (teacher-student and peer relationships), the teaching methodology and different organizational components (inadequacy of study plans, scheduling problems, overlapping programs, low student participation in management and decision-making, overcrowding, etc.) (35–37). Deane and Song (38), in a study that described eleven potential situations that generate stress and stress symptoms, found that the situations most predictive of chronic stress were class participation, required assignments and test taking. Bob et al. (39), in a sample of medical students, found that the top stressors were exams, falling behind in the learning schedule, a large amount of content to be learned, heavy workload, and lack of time to review what had been learned.

#### Academic Stress Symptoms

The stress response refers to the physiological, emotional or behavioral manifestations triggered by stressors (40). An acute stressor can trigger various physiological responses (rapid heartbeat, blood pressure, increased respiratory rate and corticosteroid levels, sweating, trembling, headaches, weight loss or gain, body aches, sleep quality issues). It can also affect the subjective experience in relation to cognitive reactions (perceived stress, negative thoughts, worry, sense of uncontrollability) and negative affect (irritability, agitation, fear, anxiety, guilt) (41) as well as generate behavioral responses (crying, abuse of self and others, smoking) (12, 42–44).

Previous research has shown that high levels of stress are associated with problems of physical health (45), ability to self-regulate (10) poor adjustment to college (46) and poor achievement (47, 48), involvement in unhealthy behaviors (7), depression (49), reduced well-being (33, 50) and less life satisfaction (51).

### Personality: Big Five Model

The Big Five (BF) personality traits, also known as the Five Factor Model (FFM) and the OCEAN Model (52), represents the most commonly used personality framework in current psychological literature. Although not universally accepted (52, 53), there is general agreement in the prior research on personality that the BF taxonomy describes the basic personality dimensions that have a substantial genetic basis (54). A great deal of empirical support has shown its universality across genders and widely differing contexts (55, 56).

The Big Five personality domains are: Openness (O), Conscientiousness (C), Extraversion (E), Agreeableness (A) and Neuroticism (N) (53). Openness implies the tendency to be imaginative, curious, flexible and insightful. *Conscientiousness* has to do with individual differences in the manner of focusing on tasks and bringing them to completion; four facets of C consistently appear in many research studies: orderliness, industriousness, responsibility and self-control. Several non-cognitive constructs widely used in education are very similar to C, specifically, grit, time management, motivation, self-regulation, performance/mastery goals, and mindset. *Extraversion* describes the tendency to be outgoing, assertive and energetic and to show positive emotionality. *Agreeableness* has to do with being friendly, cooperative and generous, altruistic, modest, compassionate. However, *Neuroticism* (vs. emotional stability) implies the tendency to show anxiety, worry, negative emotionality, vulnerability, self-consciousness, physiological reactivity to stress, and behavioral inhibition (57, 58).

Recent research in the University context shows that C is the most closely related factor to academic performance; it encourages intrinsic academic motivation, prevents procrastination, and predicts high achievement (52, 59, 60). Schneider and Preckel's (61) systematic review of 38 meta-analyses found that, among 16 personality variables related to academic achievement, Conscientiousness showed the largest absolute effect size in predicting academic performance.

In adulthood as well, empirical studies show an association between personality traits and mental well-being. Kokko et al. (62), in their review, indicate that Neuroticism is linked most closely (and negatively) to different aspects of emotional well-being, including happiness, life satisfaction, affectivity, and quality of life. Steel et al. (63), in their meta-analysis of 347 samples, found that the Big Five personality traits played a very important role in emotional well-being, accounting for 40 to 60 per cent of its variance.

#### The Graded Sequence of Self-Regulation in the Big Five Factors

Recent research has attempted to establish the possibility of a general, transdiagnostic *p* factor, referring to the lack of self-regulation of goal pursuit (64). In a complementary approach, the theoretical model of *SR vs. ER Theory* (65) has hypothesized the existence of a continuum of self-regulatory, non-regulatory and dysregulatory behaviors in persons. This theory has contributed recent evidence for three claims: (1) students' levels of behavior self-regulation (high-medium-low) determine corresponding levels in several affective-motivational variables, such as engagement, resilience, and stress responses (66); (2) these regulation levels (high-medium-low) are interdependent with self-regulation/non-regulation/dys-regulation behaviors, respectively, and health behaviors (67); (3) regulation levels have also been found in association with personality factors (68). For these reasons, specifically, it proposes a self-regulatory ordered sequence of personality components, from greater to lesser levels of self-regulation behavior, namely: *Conscientiousness* and *Extraversion* (self-regulatory components), *Openness to Experience and Agreeability* (non-regulatory components), and *Neuroticism* (dysregulatory component). The first two would be associated with a greater self-regulatory component (consistent pattern of proactive self-regulation behaviors, especially prominent in C), the next two would be non-regulatory in nature (not consistently proactive, more reactive to the regulatory input of the context), and finally neuroticism, with a dysregulatory nature (consistent pattern of self-induced lack of self-regulation). In this regard, evidence has been presented with reference to a possible gradation of the Big Five model factors, based on relations between each component and its accompanying level of self-regulation (68, 69).

### Resilience

Most researchers agree on the general definition of *resilience* as the ability to withstand adversity and recover from stress and negative experiences (70). Expanding on this definition, it can be said that resilience is also the ability to advance and grow in response to difficulties and challenges, that is, to find strength through adversity (71). Resilience is not limited to people with traumatic experiences or minority groups, it appears to be generally beneficial for study progress and for dealing with the typical challenges of University contexts (19).

Prior evidence points to resilience as a *key skill for students* (72), positively associated with academic engagement, academic persistence (4), mental health, well-being (73), and self-regulation (74, 75).

#### Reactive and Proactive Components of Resilience

Recent research has suggested the existence of two types of dimensions or types of factors that make up resilience, based on the CD-RISC scale (76), that is, *reactive* and *proactive* components of resilience. On one hand, there are the behavioral factors of resilience pertaining to endurance in the face of adverse conditions (reactive factors); on the other hand is the ability to bounce back and produce changes in the conditions that caused the adverse situation (proactive factors). In the first case, stress management skills and spirituality behaviors have been shown to predict emotion-focused coping strategies; in the second case, perceived competence, the ability to adapt to change and perceived control (self-regulation) have been shown to predict problem-focused strategies. Consequently, the two dimensions are complementary and necessary, although only the proactive factors would pertain to self-regulatory behavior (76).

### Relationships Between the Big Five, Resilience, and Perceived Stress (Factors and Symptoms)

#### BF and Resilience

Earlier research has identified a link between all five dimensions of personality and a person's ability to bounce back. In his study, Grossman (77) analyzed if the utility of resilience as a construct to predict certain criteria (measures of physical health, mental health, and well-being) is greater than that of established classical predictors, such as personality traits (BF model). Generally speaking, their results lent support to the hypothesis that general resilience was positively correlated (at least moderately) with all the BF traits. Extraversion showed the lowest correlation (0.48) and Conscientiousness showed the highest (0.64). Agreeableness showed only a moderate to low correlation with general resilience (0.40) at the 95% confidence interval. The Grossman meta-analysis suggest that resilience overlaps substantially with big-five prersonality traits and offer limited utility above personality in predicting health or well-being outcomes. In another meta-analysis (78), results also indicate specifically that resilience is positively related to Extraversion, Openness, Agreeableness, Emotional stability and Conscientiousness (close to 0.40) and negatively to Neuroticism (−0.46). One of the most frequently used scales in these studies is the CD-RISC (79).

The sphere of health sciences is where much attention has been given to relations between resilience and the Big Five (80, 81). The conceptual *model of medical student well-being* (82) points to personality and temperament factors as fundamental to resilience (83). Recent findings also showed that all resilience factors were positively correlated with the personality profiles of well-adjusted individuals. Significant, positive relations were found between dimensions of resilience and personality traits E, O, A, and C; and negative relations were found with neuroticism (19, 84, 85).

The traits most closely related to resilience were Extraversion, Conscientiousness and Neuroticism (86, 87). In the face of challenges, Conscientious students were able to make structured action plans, thereby building resilience and enabling them to progress in their studies (19). Pendergast (88) points to the strong relationship between Conscientiousness and Resilience as something to be expected, since C in college students may buffer against stress; but he also notes that this strong relationship appears when is used the CD-RISC scale, so it is possible that there is certain overlap between the two constructs. In fact, certain items that address the tenacity factor of CD-RISC may be measuring some aspect of Conscientiousness. On the other hand, Extraversion and also Agreeableness are associated with good social skills that encourage cooperation and social support that is an important protector of resilience (19).

In general, prior research may be said to show that resilient students possess a repertory of personality traits (particularly C and E) and coping styles (problem-focused) that act as internal protective factors, allowing them to better adapt to difficulties and stress (19).

#### BF and Perceived Stress

Research shows that people with different personality traits manifest different reactions to stress, which in turn affect their adjustment at University. There is substantial evidence about the N factor is the main predictor of high levels of subjective stress experience; less is known about how the other four factors relate to stress, and findings are less consistent (13, 43). For some time, there was little analysis of how all the Big Five factors were related to stress. Such studies are now increasing (14), including recent papers that examine the Big Five alongside the biological and physiological correlates of stress (43).

Recent research generally corroborates these findings, with certain qualifications. Xin et al. (43) indicate that N, E and O are important variables associated with the stress response, and that different dimensions of personality are associated with different aspects of the stress response. Their data indicate that higher Neuroticism predicted the physiological stress response (heartbeat and cortisol activation), a bigger drop in positive affect and lower subjective control capacity. They also indicated that individuals with greater Extraversion showed less cortisol activation to stress, and a good, resilient psychological response, with less increase in negative affect. A higher score in Openness was also associated with less cortisol response to stress. However, they point to certain inconsistencies between studies that can be attributed to factors like the subjects' demographic variables, the studies analyze different stressors, they use different measurements of the stress response, and there are issues of scientific bias. Soliemanifar et al. (89), as well as Xin et al. (43), have also analyzed the causal link between BF dimensions and biological aspects (cardiovascular and endocrine response to stress); they consider that the BF model provides a psychobiological typology of stress reactivity.

The connection between basic personality factors of Neuroticism, Extraversion, and Conscientiousness, stress experience and coping (18), seems well-established. Certain studies indicate that O and A have weaker or null association with stress (43). However, results about relationship between C and stress are still inconsistent. Certain studies have found that students with high levels of Conscientiousnness are less resistant to stress (13).

In general, the literature recognizes the role of personality traits in academic stress (90), academic performance (91) and coping (58). High neuroticism pre-disposes students to stress, making them more vulnerable. Extraversion, Conscientiousness and also Agreeableness can act as protective factors under stressful conditions. Thus, we can assume that a student with a high level of Neuroticism will consider a stressful task to be a threat (the demands of the stressor are too high when compared to the coping resources), and will increase worry about one's academic skills, negative emotional response, fear of failure or fear of poor performance. On the other hand, students with high levels of E and C (moderate level) will probably assess the stressful task as a challenge, will present active coping, support seeking, avoidance of interpersonal conflict, high motivation and feelings of competence that favor good task performance.

#### Resilience and Perceived Academic Stress

Data on the role of resilience in protecting against perceived stress in undergraduate students are still limited (1), but there are some significant findings in support. Pidgeon et al. (92), in a study with an international sample of University students, found that students with low levels of resilience reported significantly lower levels of perceived social support, connectedness on campus and higher levels of psychological anxiety, compared to University students with high levels of resilience Hernandez et al. (47) also found that higher levels of academic stress were associated with less ability to bounce back. In this study, the group classified as not resilient had higher stress scores, lower self-efficacy and slightly lower academic achievement than the group classified as resilient. Pidgeon and Pickett (93), with a sample of University students with high and low levels of resilience, reported that students in the low resilience group experienced significantly lower levels of mindfulness, higher levels of psychological distress, more limited use of adaptive coping, and greater use of maladaptive coping, in comparison to students with high levels of resilience (46, 94, 95), in 4-year longitudinal studies with University students examine multiple aspects of psychosocial adjustment: (a) psychological functioning (self-esteem and psychological distress, that is, depression, anxiety and stress), (b) cognitive-affective strategies (including active and avoidant emotional coping), and (c) social well-being (specifically social support from friends) with the purpose of identifying change patterns of risk and resilience. Their results indicate that adjustment over the 4 years at University did not change in linear fashion. Student adjustment generally worsens across the first 2 years followed by some improvement in the last two, although only self-esteem and active emotional coping were completely recovered in women, and only the latter in men.

#### BF, Resilience, and Perceived Academic Stress

Resilience and vulnerability to stressors depend on age, sex, intelligence, and many other personality characteristics. Lecic-Tosevski et al. (96) indicate that the relationship between personality and stress has an impact on four important aspects: (1) choice or avoidance of settings that are associated with specific stressful factors, challenges or benefits, (2) the way one interprets a stressful situation and assesses one's own skills and abilities for adopting a proactive attitude and behavior to either confront or avoid it, (3) intensity of one's response to a stressor, and (4) coping strategies used by the individual facing a stressful situation.

Today there is a growing interest in understanding the relations between resilience, personality traits and stress (28). However, there are relatively few studies that analyze the mediational role of resilience between the Big Five and stress responses at University (30).

Some have analyzed the mediational role of resilience between personality and happiness (97) or depressive symptoms (29). For their part, Sarrionandia et al. (1) have analyzed resilience as a mediator of emotional intelligence and perceived stress. Backman et al. (19) have studied the role of the Big Five personality dimensions and Resilience in students' achievement and study progress. Their results show that four of the five dimensions of the BF model, specifically Openness, Conscientiousness, Emotional Stability and Extraversion, were related to Resilience, and Resilience in turn resulted in better study progress. Their results of the mediation analyses also show that introducing resilience does not decrease the predictive validity of the Big Five. On the other hand, they found that Openness was negatively related to study progress, while positively related to resilience. The results of how Neuroticism relates to study progress were not clear. A significant relationship between emotional stability and study progress was not supported, but they found that emotional stability can help foster study progress through student's ability to bounce back.

The study by Shi et al. (30) is one of the few to examine the relationship between personality traits (BF) and anxiety symptoms among medical students and the first to study the mediational role of resilience in this relationship. Their results indicate that A, C and O were negatively associated with anxiety while N was positively associated. They found that resilience works as a mediator of the relationships between A, C, O and anxiety symptoms. The authors conclude that identifying individuals at risk and implementing intervention strategies focused on personality traits and resilience can be an effective strategy to prevent and reduce anxiety symptoms.

### Present Study: Aims and Hypotheses

Despite ample prior evidence on the constructs of the BF model and Resilience, no specific evaluation has been made of predictive relationships of the personality factors (according to their regulatory nature) for resilient behavior (in its reactive and proactive components). Nor has the specific mediating role of resilience been analyzed in its relation to academic stress factors from the teaching-learning process, and to symptoms of academic stress. Therefore, the aim of this study was to establish these predictive relations. The following affirmations were hypothesized:

#### Hypothesis 1

The more *regulatory* factors of the BF model (C and E) will significantly and positively predict *total resilience*, as well as proactive factors of resilience (perceived competence, adaptation to change, and perceived control); the *non-regulatory* factors of the BF model (O, A) will not be predictive of the proactive factors but will be more predictive of reactive factors of resilience, such as stress management and spirituality; finally, the *dysregulatory* factor of the BF (N) will prove to be a significant, negative predictor of total resilience and of its reactive factors. This predictive scheme will be maintained in regard to the factors and symptoms of academic stress; thus, while C and E will negatively predict the factors and symptoms of academic stress, factors O and A will have a neutral relationship and factor N will predict them positively.

#### Hypothesis 2

The proactive factors of resilience (perceived competence, adaptation to change and perceived control) will significantly, negatively predict stress factors and symptoms; however, reactive factors of resilience (endurance of stress and spirituality) will be non-significant predictors of such stress factors and symptoms.

#### Hypothesis 3

Stress factors from the process of teaching and learning (especially the latter) will be the strongest predictors, positively and significantly, of stress symptoms in students.

#### Hypothesis 4

The general structural prediction model will show that the different personality factors, in conjunction with the different resilience factors, will be significant, negative predictors of stress factors and symptoms. In the case of the different BF and resilience factors, there will be corresponding indirect effects, similar to those referenced (positive, neutral or negative) in the stress factors and symptoms.

#### Hypothesis 5

The specific mediational models for each BF factor will significantly show a positive mediational value of resilience for predicting stress factors and symptoms in students, based on whether the personality factors are *regulatory* (C, E), *non-regulatory* (O, A), or *dysregulatory* (N).

## Methods

### Participants

The study sample contained an initial 665 undergraduate students selected from two universities in Spain. These students were pursuing degrees in Psychology, Primary Education, and Educational Psychology; 85.5% were women and 14.5% were men. After confirming significant gender differences in the variables, we limited our sample to the 405 female students. Age range was 19–25, with an average age of 21.33 years. The study design was incidental and non-randomized. The Guidance Department at each University invited teacher participation, and the teachers invited their own students to participate on an anonymous, voluntary basis. Each course (subject) was considered one specific teaching-learning process; questionnaires were completed online for each subject.

### Instruments

The *Big Five Questionnaire*, BFQ-N (98), based on Barbaranelli et al. (99). The adaptation used in this study was for young University students (32). Confirmatory Factor Analysis (CFA) reproduced a five-factor structure corresponding to the Big Five Model. Results showed adequate psychometric properties and acceptable fit indices. The second-order confirmatory model showed good fit [Chi-square = 38.273; Degrees of freedom (20–15) = 5; *p* < 0.001; NFI = 0.939; RFI = 0.917; IFI = 0.947; TLI = 0.937, CFI = 0.946; RMSEA = 0.065; HOELTER = 2,453 (*p* < 0.05) and, 617 (*p* < 0.01)]. The total scale showed good internal consistency (Alpha = 0.956; Part 1 = 0.932, Part 2 = 0.832; Spearman-Brown = 0.962; Guttman = 0.932).

#### Resilience

Measured using the CD-RISC Scale (100) in its validated Spanish version (101, 102). This scale assesses different aspects of how one faces difficulties and is able to overcome them. The results offer information on perception of competence, acceptance of change and secure relationships, tolerance/stress management, control and spirituality (103). Adequate reliability and validity values were obtained in Spanish samples, and a five-factor structure: F1. Persistence/tenacity, strong self-efficacy (COMPETENCE); F2: Emotional and cognitive control under pressure (STRESS); F3: Adaptability/ability to bounce back and secure relationships (CHANGE); F4: Perceived Control (CONTROL), and F5: Spirituality (SPIRITUALITY).

#### Factors of Stress

*Cuestionario de Estrés Académico, CEA* [Academic stress questionnaire] (35, 104). The scale's internal structure was analyzed. In order to do so, we conducted a confirmatory factor analysis (CFA) of the whole set of data from our sample, thus verifying the second-level structure. The default model has good fit [Chi-Square = 66,457, df = 13, *p*< *0*.001; CFI = 0.935, TLI = 0.961, IFI = 0.947, RFI = 0.965 and NFI = 0.947; RMSEA = 0.057; HOELTER = 0.430 (*p* < 0.05) and 0.532 (*p* < 0.01)]. The proposed model contains 53 items with a seven-factor structure having two dimensions, where one factor differs from the original version. The resulting dimensions and factors were: (1) *Dimension of Stress in Learning:* Task Overload (Factor 2), Dificulties of Performance Control (F3), Social climate (Factor 5), and Test Anxiety (Factor 7); (2) *Dimension of Stress in Teaching*: Methodology difficulties (Factor 1), Public speaking (Factor 4); Content lacks value (Factor 6). Overall reliability = 0.961; part 1 = 0.932, part 2 = 0.946.

#### Effects of Stress

*Stress Response Questionnaire, CRE* (105). The scale's psychometric properties were found to be adequate in this sample of Spanish students. The confirmatory structural model of the CRE has the following dimensions (Chi-square = 846,503; Degrees of freedom (275 – 76) = 199, *p* < 0.001; NFI = 0.952; RFI = 0.965; IFI = 0.953): F1. *Burnout*; F2. *Sleep Difficulties*; F3. *Irritability;* F4. *Negative thoughts*; F5. *Agitation*. Scale unidimensionality and metric invariance were confirmed in the assessment samples [RMSEA = 0.046; CFI 0.922 and TLI 0.901; HOELTER = 431 (*p* < 0.05) and 459 (*p* < 0.01)]. Cronbach alpha was 0.920, part 1 = 0.874 and part 2 = 0.863.

### Procedure

Research participants received information about this research study and gave their informed consent online, through the Academic Stress e-Coping platform (106), in the context of a more extensive research project (R&D Project ref. 2019–2021). For more detail, see http://www.inetas.net.

The questionnaires were completed by students on a voluntary basis. Data were collected and processed with the students' informed consent, in accordance with the Ethical and Deontological Principles of Psychology. The data were handled anonymously, in a group format, and were stored in a protected database at the University. The Bioethics Committee approved the Project and the instruments used (ref. 2018.170).

### Data Analysis

Using an *ex post* facto, transversal design (107), we performed three types of analyses. The usual assumptions of regression analysis were tested beforehand.

#### Preliminary Analysis

First, we explored the quality of the data by testing for outliers and missing cases. We tested for *univariate* outliers by calculating the typical scores of each variable, considering cases with Z scores outside the ±3 range to be potentially atypical cases (108, 109). On the other hand, the Mahalanobis distance (D2) was used to detect atypical *combinations* of variables (atypical multivariate cases), a statistical measure of an individual's multidimensional distance from the centroid or mean of the given observations (107). This procedure detects significant distances from the typical combinations or centroids of a set of variables. The literature suggests removing univariate and multivariate outliers, or reassigning them the nearest extreme score (110). The procedure was carried out using SPSS (v.26, IBM, Armonk, NY, USA), which provides a specific routine for missing values analysis that determines the magnitude of missing values and whether they are presented in a systematic or random manner.

Assumptions related to sample size, independence of errors, univariate and multivariate normality, linearity, multicollinearity, recursion, and interval measurement level were also evaluated, and represented acceptable reliability levels. Regarding the sample size, inclusion of 10–20 cases per parameter is recommended, and at least 200 observations (111).

Independence of errors means that the error term of each endogenous variable must not be correlated with other variables. In order to test for univariate normality, we examined the distribution of each observed variable, and its indices of asymmetry and kurtosis. Asymmetry values >3 and kurtosis >10 suggest that the data should be transformed. On the other hand, values <70 on the Mardia multivariate index indicate that distance from the multivariate normality is not a critical deterrent to this analysis. Although one of the assumptions is level of interval measurement, in some cases, variables measured at a nominal or ordinal level were used, as long as the distribution of scores, particularly of the dependent variables, was not markedly asymmetric.

As a preliminary analysis, we checked for normal sample distribution using the Kolmogorov-Smirnoff test for dependent variables. We also used the Hoelter Index to test for adequate sample size (75). In addition, we conducted analyses of linearity and atypical values, missing and influential cases, as well as critical values of multivariate normality; recommended values for the multivariate index of kurtosis, or Mardia coefficient, were <0.70 (112).

#### Predictive Analysis

We applied multiple regression analysis, using SPSS (v.25), for Hypotheses 1–3.

#### Structural Prediction and Mediational Models

Hypotheses 4 and 5 were tested using a Structural Equation Model (SEM) and mediational model, for complex measurement (113). We assessed model fit by first examining the ratio of chi-square to degrees of freedom, then the Comparative Fit Index (CFI), Normed Fit Index (NFI), Incremental Fit Index (IFI), and Relative Fit Index (RFI). All fit measures of the incremental model were above the suggested limit of 0.90 (114): Comparative Fit Index (CFI), Incremental Fit Index (IFI), Normed Fit Index (NFI), Relative Fit Index (RFI) and Tucker-Lewis Index (TLI). The value of the Comparative fit index (CFI) was equal to 0.928, which is also satisfactory. We replicated the results of the original scale. The value of the Root Mean Square Error of Approximation (RMSEA) was 0.084, less than the warning value of 0.09 (115). These should ideally be >0.90. We also used the Hoelter Index to determine adequacy of sample size. AMOS (v.22) was used for these analyses. Keith (116) proposed the following beta coefficients as research benchmarks for *direct effects*: < 0.05 is considered too small to be meaningful, above 0.05 is small but meaningful, above 0.10 is moderate, and above 0.25 is large. For *indirect effects*, we used Kenny's definition (117) of an indirect effect as the product of two effects; using Keith's benchmarks above, we propose a small indirect effect = 0.003, moderate = 0.01, and large = 0.06, values that are significant in the sphere of education.

## Results

### Preliminary Analyses: Normality Assumptions

Results from the analyses used to test normality, a prerequisite for linear analysis, showed adequate distribution of sample variability. See Table 1.

**Table 1:** Descriptive values of the study variables (*n* = 405).

**Variable**	**Min**	**Max**	**M (sd)**	**Mean std. error**	**Asymmetry**	**Standard asymmetry error**	**Kurtosis**	**Standard kurtosis error**	**Kolmogoroff-Sminoff**
C	1.92	5.00	3.733 (0.566)	0.025	−0.138	0.109	−0.288	0.217	0.037. *p >* 0.200
E	1.23	4.92	3.592 (0.560)	0.025	−0.517	0.109	0.557	0.217	0.048. *p* > 0.112
O	1.92	4.85	3.496 (0.474)	0.021	0.018	0.110	−0.003	0.220	0.041. *p >* 0.132
A	2.46	5.00	4.024 (0.474)	0.021	−0.280	0.109	−0.316	0.218	0.026. *p >* 0.215
N	1.08	5.00	2.616 (0.635)	0.028	0.255	0.109	0.120	0.217	0.050. *p* > 0.120
Resilience	1.82	4.86	3.760 (0.016)	0.016	−0.573	0.089	0.576	0.178	0.054. *p >* 0.100
Stress factors	1.29	4.52	2.993 (0.032)	0.045	−0.166	0.059	−0.175	0.122	0.042. *p* > 0.200
Stress symptoms	1.04	5.00	2.308 (0.056)	0.031	0.487	0.107	0.425	0.215	0.045. *p >* 0.150

*C, Conscientiousness; E, Extraversion; O, Openness to Experience; A, Agreeability; N, Neuroticism*.

### Specific Linear Prediction Relationships Between the Big Five Factors and Resilience (Hypothesis 1)

Regression analyses showed that the personality factors had differential predictive value for Resilience factors. The regulatory personality factors (C and E) significantly and positively predicted total resilience, as well as proactive resilience factors (competence, change and control), but this was not so for reactive factors (stress management and spirituality). C and E most strongly predicted Perceived competence, followed by Perceived control. The non-regulatory personality factors (O and A) did not generally predict total resilience, although they were significant predictors of certain reactive resilience factors, positively predicting stress management and negatively predicting spirituality. The dysregulatory personality factor (N) negatively predicted total resilience and most of its factors, except for spirituality, where it had no predictive power. See Table 2.

**Table 2:** Multiple regression between the BF factors and Resilience (*n* = 405).

**Big five**	**Competence**	**Stress mgmt**	**Change**	**Control**	**Spirituality**	**Total**
C	0.310^**^	0.001	0.165^*^	0.321^**^	0.097	0.261^**^
E	0.297^**^	0.214^**^	0.265^**^	0.242^**^	0.098	0.276^**^
O	0.063	0.260^**^	0.144^*^	−0.032	−0.123^*^	0.051
A	−0.087	−0.082	−0.04	0.002	0.122^*^	0.045
N	−0.298^**^	−0.254^**^	−0.268^**^	−0.222^**^	−0.061	−0.210^**^
**F**_(5, 415)_	46.991^**^	22.402^**^	33.487^**^	33.496^**^	4.559^**^	23.529^**^
Adj. **R**^2^	0.376	0.239	0.279	0.314	0.052	0.319

*C, Conscientiousness; E, Extraversion; O, Openness to Experience; A, Agreeability; N, Neuroticism*.

* *p < 0.05*,

** *p < 0.01*,

*** *p < 0.001*.

### Linear Predictive Relationships of the Big Five, for Factors and Symptoms of Academic Stress (Hypothesis 1)

Results from the regression analysis offered interesting clarifications. The regulatory personality factors (C and E) were significant, negative predictors of total factors of academic stress, especially factors pertaining to the learning process (overload, achievement control). Non-regulatory personality factors (O, A) were mixed predictors of stress factors (O negatively and A positively). Worthy of note is that factor A was a significant, positive predictor of stress factors from the teaching. The dysregulatory personality factor showed the greatest predictive power (*B* = 0.403; *p* < 0.001), as a significant, positive predictor of total stress factors, most noticeably of lack of control over achievement (*B* = 0.437; *p* < 0.001).

The same tendency was repeated in the prediction of stress symptoms. The regulatory personality factors (C and E) were significant, negative predictors of total symptoms of academic stress, especially of burnout and negative thinking. The non-regulatory personality factors (O, A) did not show significant predictive power on total stress factors but were differential predictors of certain specific factors. The dysregulatory personality factor was a significant, positive predictor of total stress symptoms, with the greatest power (*B* = 0.564; *p* < 0.001), where irritability was most noteworthy (*B* = 0.638; *p* < 0.001). See Tables 3, 4.

**Table 3:** Multiple regression between the BF factors, and factors of academic stress (*n* = 405).

**Big five**	**Method diff**.	**Public spkg**.	**Content value**	**Overload**	**Soc. climate**	**Low Achievemt control**	**Teaching factors**	**Learng. factors**	**Acad. stress factors**
C	−0.054	0.007	−0.034	−0.201^**^	−0.023	−0.167^**^	−0.026	−0.156^**^	−0.118^*^
E	−0.150^**^	−0.245^***^	0.012	−0.200^**^	−0.035	−0.142^*^	−0.165^**^	−0.174^**^	−0.202^**^
O	0.003	−0.242^***^	−0.002	−0.052	0.101	−0.038	−0.135^*^	−0.024	**−0.051**
A	0.195^***^	0.129^***^	0.030	0.052	−0.026	0.167^**^	0.124^*^	0.126^*^	0.152^**^
N	0.333^***^	0.302^***^	0.212^***^	0.139^***^	0.215^***^	0.437^***^	0.380^***^	0.361^***^	0.403^***^
*F*_(5, 367)_	10.751^**^	23.087^***^	4.318^**^	20.858^**^	4.273^**^	24.230^**^	17.060^***^	15.571^**^	18.557^***^
*R*^2^	0.180	0.216	0.052	0.219	0.053	0.248	0.196	0.178	**0.228**

*C, Conscientiousness; E, Extraversion; O, Openness to Experience; A, Agreeability; N, Neuroticism*.

* *p < 0.05*,

** *p < 0.01*,

*** *p < 0.001*.

*Bold values indicate major effects*.

**Table 4:** Multiple regression between the BF factors, and symptoms of academic stress (*n* = 405).

**Big five**	**Burnout**	**Sleep diff**.	**Irritability**	**Neg. thoughts**	**Restlessness**	**Academic stress symp**.
C	−0.223^**^	−0.010^*^	−0.019	−0.125^**^	−0.044	–0.111^*^
E	−0.165^*^	−0.129^*^	−0.162^***^	−0.309^***^	−0.077	–0.204^**^
O	−0.031	0.106^*^	0.015	−0.083	−0.022	–**0.014**
A	0.158^**^	−0.030	−0.114^**^	0.169^**^	0.055	**0.074**
N	0.432^***^	0.408^***^	0.638^***^	0.456^***^	0.391^***^	0.564^***^
*F*_(5, 400)_	32.338^**^	18.787^**^	72.615^***^	42.482^**^	15.617^**^	47.648^***^
*R*^2^	0.278	0.176	0.460	0.347	0.153	**0.389**

*C, Conscientiousness; E, Extraversion; O, Openness to Experience; A, Agreeability; N, Neuroticism*.

* *p < 0.05*,

** *p < 0.01*,

*** *p < 0.001*.

*Bold values indicate major effects*.

### Linear Predictive Relationships of Resilience Components for Factors/Symptoms of Academic Stress (Hypothesis 2)

Regression analyses showed differential predictive values. The proactive factors of resilience (adaptation to change, perceived control) were significant, negative predictors of the level of total stress. Specifically, the proactive factors (competence, change, control) had the greatest negative predictive power for academic stress factors, especially regarding work overload and achievement control. However, the reactive factors had less predictive power, and in the case of spirituality, there was even positive prediction of stress factors (public speaking, overload, achievement control), thereby confirming its reactive, stress-enduring value.

In complementary manner, this tendency was repeated for stress symptoms. The proactive factors mentioned (competence, change and perceived control) were significant, negative predictors of stress symptoms, while the reactive factors (stress management, spirituality) were not so. Worth mentioning was spirituality as a negative predictor of burnout, indicating the buffering role of this factor. See Tables 5, 6.

**Table 5:** Multiple regression between the factors of resilience and factors of academic stress (*n* = 405).

**Resilience**	**Method diff**.	**Public spkg**.	**Content value**	**Overload**	**Soc. climate**	**Achievemt control**	**Teaching factors**	**Lrng. factors**	**Acad. stress factors**
Competence	0.054	−0.085	−0.027	−0.096	0.001	−0.158^**^	−0.022	−0.070	–**0.027**
Change	−0.138^*^	−0.176^**^	−0.095	−0.169^**^	−0.045	−0.058	−0.172^*^	−0.232^**^	–0.232^**^
Control	−0.067	−0.059	−0.092	−0.091^*^	−0.102	−0.139^**^	−0.112^*^	−0.110	–0.138^*^
Stress	−0.114	−0.136^*^	0.024	−0.056	0.004	−0.058	−0.076	−0.040	–**0.022**
Spirituality	0.033	0.099^*^	−0.019	0.079^*^	0.093^*^	0.129^**^	0.063	0.085	**0.071**
*F*_(5, 392)_	2.385^**^	16.793^**^	3.383	16.585^**^	3.642^*^	15.944^**^	10.234^**^	10.753^**^	11.990^**^
*R^2^*	0.025	0.143	0.033	0.120	0.028	0.086	0.101	0.113	**0.113**

*Competence, Perceived competence; Change, Adaptation to change; Control, Perceived Control; Stress, Stress management*.

* *p < 0.05*,

** *p < 0.01*,

*** *p < 0.001*.

*Bold values indicate major effects*.

**Table 6:** Multiple regression between factor of stress, and symptoms of academic stress (*n* = 405).

**Resilience**	**Burnout**	**Sleep diff**.	**Irritability**	**Neg. thoughts**	**Restlessness**	**Stress symptoms**
Competence	−0.195^**^	−0.069	−0.168^**^	−0.313^**^	−0.091	–0.187^*^
Change	−0.069	−0.107	−0.069	−0.156^*^	−0.080	–0.126^*^
Control	−0.139^**^	−0.114^**^	−0.126^**^	−0.051	−0.093^*^	–0.122^*^
Stress	0.021	0.027	−0.042	−0.028	−0.004	–**0.023**
Spirituality	0.081^*^	−0.019	−0.020	0.056	−0.017	–**0.054**
*F*_(5, 382)_	16.317^**^	8.093^**^	17.322^**^	40.067^**^	7.500^**^	21.557^**^
*R*^2^	0.110	0.051	0.111	0.228	0.054	0.152

*Competence, Perceived competence; Change, Adaptation to change; Control, Perceived Control; Stress, Stress management*.

* *p < 0.05*,

** *p < 0.01*,

*** *p < 0.001*.

*Bold values indicate major effects*.

### Linear Predictive Relationships of Factors and Symptoms of Academic Stress (Hypothesis 3)

The directionality of the regression results consistently showed that stress factors from the learning process had the greatest predictive power on stress symptoms. The factor of loss of achievement control was especially relevant, predicting all stress symptoms. See Table 7.

**Table 7:** Multiple regression between factor of stress, and symptoms of academic stress (*n* = 405).

**Stress factors of teaching**	**Burnout**	**Sleep diff**.	**Irritability**	**Neg. thoughts**	**Restlessness**	**Stress symptoms**
Method diff.	0.077	0.068	0.038	−0.063	−0.013	**0.033**
Public spkg.	0.108^**^	0.001	0.058	0.129^*^	0.025	**0.070**
Content value	0.064	−0.98	0.036	−0.076	−0.078	–**0.034**
Overload	0.282	0.075	0.123^*^	0.130^*^	0.048	0.156^**^
Social climate	−0.008	0.098	0.126^**^	0.020	0.181^*^	0.115^**^
Loss of achievement control	0.184^**^	0.305^**^	0.249^**^	0.466^**^	0.344^**^	0.339^**^
*F*_(5, 382)_	40.217^**^	17.923^**^	26.767^**^	40.536^**^	22.983^**^	50.775^**^
*R*^2^	0.322	0.183	0.241	0.326	(0.215)	**0.401**

* *p < 0.05*,

** *p < 0.01*,

*** *p < 0.001*.

*Bold values indicate major effects*.

### Structural Prediction Model (Hypothesis 4)

Two structural models were tested. The first model took only the BF factors as independent variables. The second model–which attained greater statistical significance–took both BF and Resilience as criterion variable. All these measures were indicative of good model fit. See Table 8.

**Table 8:** Models of structural linear results of the variables.

**Model**	**Degrees of freedom**	**Chi-square**	***p < *.**	**NFI**	**RFI**	**IFI**	**TLI**	**CFI**	**RMSEA**	**Hoelter**	
										**0.005–0.001**	
1. BF	(152-51): 101	883.554	0.001	0.804	0.832	0.820	0.853	0.819	0.075	215–235	
2. BF and R	(252-67): 185	1233.657	0.001	0.905	0.956	0.929	0.959	0.928	0.053	293–359	

*Model 1: Big Five; Model 2: Big Five and Resilience*.

**Figure 1:**
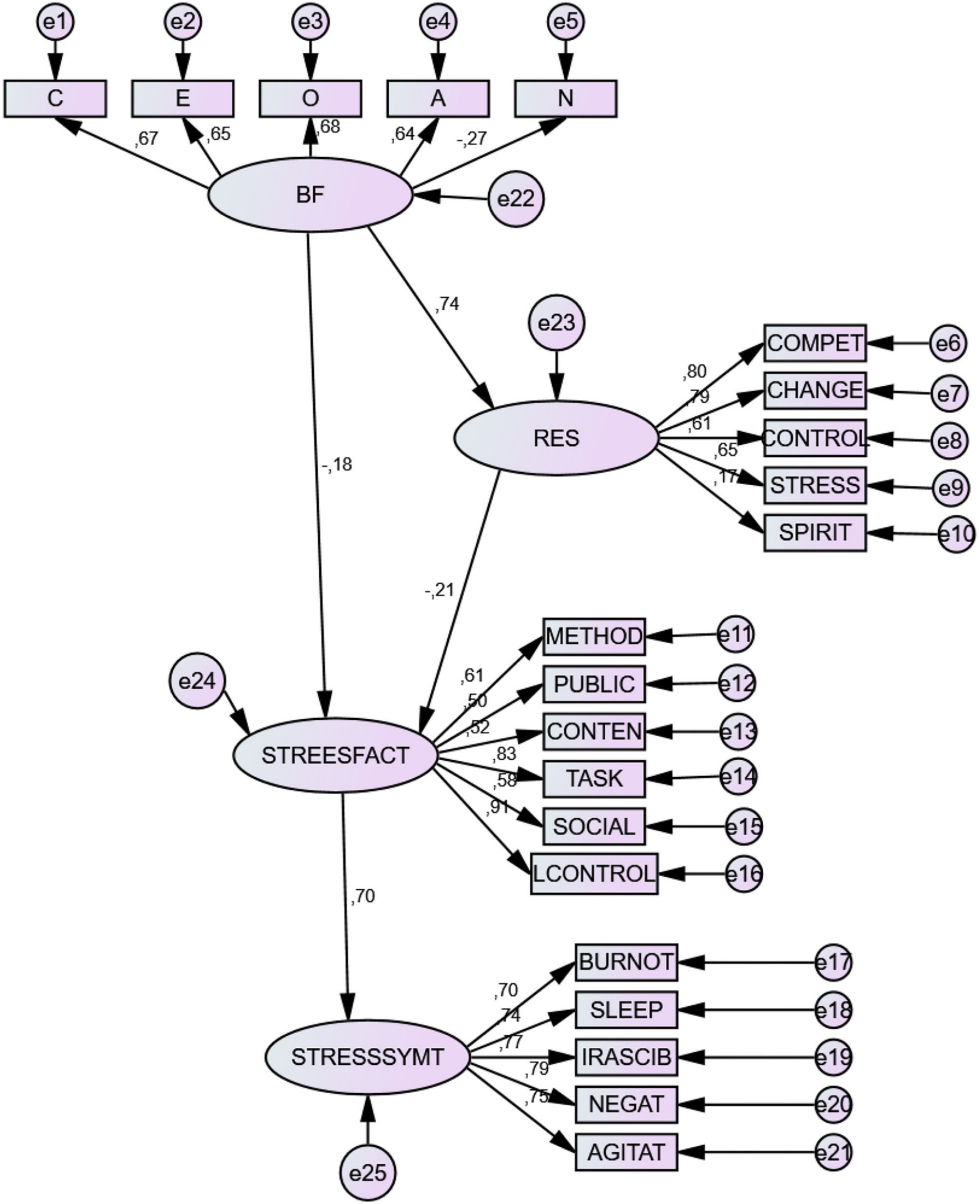
Structural Prediction of variables in the second model. E, Extraversion; C, Conscientiousness; N, Neuroticism; O, Openness to Experience; A, Agreeability; RESIL, Resilience; STRESSFACT, Stress Factors; STRESSSYMNT, Stress symptoms.

Model 2 reflected how BF factors (except for N) were positive predictors of Resilience (R), and how Stress Factors (SF) positively predicted Stress Symptoms (SS). Regarding the indirect effects of the BF Factors, these factors proved to have: (1) a positive effect on factors of Resilience; and (2) a negative effect on Stress Factors (SF) and Stress Symptoms (SS), as well as their components. In addition, resilience factors showed a negative effect on the factors and symptoms of stress. See Table 9 and Figure 1.

**Table 9:** Total, indirect, and direct effects of the variables in this study, and 95% bootstrap confidence intervals (CI).

**Predictive variable**	**Criterion variable**	**Total effect**	**CI (95%)**	**Direct effect**	**CI (95%)**	**Indirect effect**	**CI (95%)**	**Results, effects**	**CI (95%)**
BF →	Resilience	0.74	(0.69, 0.77)	0.74	(0.69, 0.77)	0.00	(−0.03, 0.02)	Direct only	(0.69, 0.74)
BF →	Stress factors	−0.34	(−0.11, −0.17)	−0.09	(−30, 38)	−0.24	(−0.22, −0.27)	Partial mediation	(−0.22, −0.27)
BF →	Stress symptoms	−0.24	(−0.20, −0.28)	0.00	(−0.20, −0.28)	−0.24	(−0.20, −0.27)	Full mediation	(−0.20, −0.27)
Resilience →	Stress factors	−0.34	(−31, −0.37)	−0.34	(−31, −0.37)	0.00	(−0.02, 0.02)	Direct only	(−31, −0.37)
Resilience →	Stress symptoms	−0.24	(−0.20, −0.28)	0.00	(−0.03, 0.04)	−0.24	(−0.20, −0.28)	Full mediation (suppression)	(−0.20, −0.28)
Stress factors →	Stress symptoms	0.70	(0.68, 72)	0.70	(0.68, 72)	0.00	(−0.03, 0.02)	Direct only	(0.68, 72)

*CI, confidence interval. Bootstrapping sample size = 430*.

Figure 1 shows general prediction relationships of the model. The Big Five personality factors, except for Neuroticism (N) (*B* = −0.27), are positive predictors of *resilience* (RES). The proactive components of resilience (competence, change and control), as well as its reactive components (stress management and spirituality), are negative predictors of stress factors (STRESSFACT), although spirituality shows less weight in this prediction (SPIRIT) (*B* = 0.17). The general mediating effect of resilience is thereby demonstrated. Stress factors may originate in aspects of the teaching process as well as the learning process; they are positive predictors of academic stress symptoms (STRESSSYMT). However, this general mediation model has a basic limitation, in that it cannot confirm the direct and indirect effect of each personality factor. On this account, other specific prediction models were carried out for each personality factor and resilience as a mediating variable.

### Mediational Model (Hypothesis 5)

The results from testing the five mediation models were differentially significant. Model 1 (Conscientiousness) and Model 5 (Neuroticism) showed the best fit and consistency, especially the latter. Model 2 (Extraversion), Model 3 (Openness to Experience) and Model 4 (Agreeability), appeared in the direction expected, though with lower significance. See Table 10.

**Table 10:** Mediational models for BF factors, resilience, and stress factors and symptoms.

**Model/factor**	**X2, df; *p*>**	**X2/df**	**NFI Delta1**	**RFI rho1**	**IFI Delta2**	**TLI**	**CFI**	**RMSEA**	**Hoelter *p < * 0.05**	**Hoelter *p < * 0.01**
1. C	1.567; (14-13) 1; *p < * 0.133	1.576	0.995	0.953	0.998	0.983	0.998	0.01	3,834	6,622
2. E	2.061; 1; *p < * 0.151	2.061	0.994	0.936	0.997	0.966	0.997	0.02	2,916	5,036
3. O	4.873; 1; *p < * 0.02	4.873	0.983	0.829	0.986	0.859	0.986	0.05	1,233	2,130
4. A	2.678; 1; *p < * 0.102	2.678	0.991	0.908	0.994	0.940	0.994	0.03	2,244	3,875
5. N	0.712; 1; *p < * 0.339	0.712	0.998	0.985	1.00	1.00	1.00	0.001	8,443	14,582

*C, Conscientiousness; E, Extraversion; O, Openness to Experience; A, Agreeability; N, Neuroticism*.

The specific analysis of each model shows the directionality of the predictions. Model 1 (Conscientiousness) shows resilience as a significant, positive mediating factor, between personality component C and academic stressors. Model 2 (Extraversion) also shows resilience as a positive mediating factor with regard to academic stressors. In Model 3 (Openness to experience) and Model 4 (Agreeability), resilience contributes an important mediating element to both of these factors that were less predictive of stress factors and symptoms. Model 5 shows a more consistent positive mediating role of resilience in buffering the stress-predicting effects of Neuroticism. See Table 11.

**Table 11:** Total, indirect, and direct effects of the variables in this study, and 95% bootstrap confidence intervals (CI).

**Predictive variable**	**Criterion variable**	**Total effect**	**CI (95%)**	**Direct effect**	**CI (95%)**	**Indirect effect**	**CI (95%)**	**Results, effects**	**CI (95%)**
**Model 1 (C)**									
Conscientiousness →	Resilience	0.46	(0.43, 0.49)	0.46	(0.43, 0.49)	0.00	(−0.02, 0.02)	Direct only	(0.43, 0.49)
Conscientiousness →	Academic Stress Factors	−0.24	(−0.28, −0.20)	−0.16	(−0.20, −0.13)	−0.07	(−0.10, −0.04)	Partial mediation	(−0.10, −0.04)
Conscientiousness →	Academic Stress Symptoms	−0.16	(−0.19, −0.14)	−0.03	(−0.05, −0.01)	−0.14	(−0.10, −0.19)	Full mediation	(−0.10, −0.19)
Resilience	Academic Stress Factors	−0.19	(−0.16, −0.22)	−0.19	(−0.15, −0.21)	0.00	(−0.03, 0.03)	Direct only	(−0.15, −0.21)
Resilience	Academic Stress Symptoms	−0.11	(−0.09, −0.12)	0.00	(−0.04, 0.03)	−0.11	(−0.08, −0.14)	Full mediation (supression)	(−0.08, −0.14)
**Model 2 (E)**									
Extravers →	Resilience	0.45	(0.49, 0.53)	0.45	(0.49, 0.53)	0.00	(−0.04, 0.03)	Direct only	(0.49, 0.53)
Extravers →	Academic Stress Factors	−0.18	(−0.23, −0.14)	−0.12	(−0.09, –.17)	−0.06	(−0.02, −0.09)	Partial mediation	(−0.02, −0.09)
Extravers →	Academic Stress Symptoms	−0.20	(−0.24, −0.15)	−0.10	(−0.06, −0.13)	−0.10	(−0.07, −0.14)	Partial mediation	(−0.07, −0.14)
Resilience	Academic Stress Factors	−0.13	(−0.17, −0.10)	−0.13	(−0.17, −0.10)	0.00	(−0.04, 0.05)	Direct only	(−0.17, −0.10)
Resilience	Academic Stress Symptoms	−0.07	(−0.11, −0.05	0.00	(−0.04, 0.04	−0.07	(−0.11, −0.05	Full mediation (suppression)	(−0.11, −0.05)
**Model 3 (O)**									
Open Exp →	Resilience	−0.32	(−0.36, −0.31)	−0.32	(−0.36, −0.31)	0.00	(−0.05, 0.04)	Direct only	(−0.36, −0.31)
Open Exp →	Academic Stress Factors	−0.23	(−0.26, −0.20)	−0.18	(−0.23, −15)	−0.47	(−0.49, −0.44)	Partial mediation	(−0.49, −0.44)
Open Exp →	Academic Stress Symptoms	−0.20	(−0.24, −0.16)	−0.06	(−0.02, −0.10)	−0.13	(−0.16, −0.11)	Partial mediation	(−0.16, −0.11)
Resilience	Academic Stress Factors	−0.14	(−0.19, −0.11)	−0.14	(−0.18, −10)	0.00	(−0.03, 0.03)	Direct only	(−0.18, −10)
Resilience	Academic Stress Symptoms	0.00	(−0.03, 04)	0.00	(−0.03, 04)	0.08	(−0.05, −11)	Partial mediation	(−0.05, −11)
**Model 4 (A)**									
Agreeability →	Resilience	0.38	(0.40, 0.35)	0.38	(0.40, 0.35)	0.00	(−0.04, 0.05)	Direct only	(0.40, 0.35)
Agreeability →	Academic Stress Factors	−0.08	(−0.12, −0.04)	−0.00	(−0.04, 03)	−0.07	(−0.12, −0.04)	Full mediation	(−0.12, −0.04)
Agreeability →	Academic Stress Symptoms	−0.18	(−0.22, −0.15)	−0.13	(−0.18, −0.12)	−0.04	(−0.07, −0.01)	Partial mediation	(−0.07, −0.01)
Resilience	Academic Stress Factors	−0.20	(−0.25, −0.15)	−0.20	(−0.25, −0.15)	0.00	(−0.03, 0.03)	Direct only	(−0.25, −0.15)
Resilience	Academic Stress Symptoms	−0.12	(−0.16, −0.08)	0.00	(−0.04, 0.03)	−0.12	(−0.15, −0.09)	Full mediation (suppression)	(−0.15, −0.09)
**Model 5 (N)**									
Neuroticism →	Resilience	−0.27	(−0.30, −0.24)	−0.27	(−0.30, −0.24)	0.00	(−0.02, 0.02)	Direct only	(−0.30, −0.24)
Neuroticism →	Academic Stress Factors	0.44	(0.48, 0.40)	0.41	(0.48, 0.40)	0.02	(−0.03, 0.04)	Direct only	(0.48, 0.40)
Neuroticism →	Academic Stress Symptoms	0.57	(0.60, 0.53)	0.34	(0.37, 0.31)	0.18	(0.15, 0.22)	Partial mediation	15, 0.22)
Resilience →	Academic Stress Factors	−0.08	(−0.11, −0.3)	−0.08	(−0.11, −0.3	0.00	(−0.03, 0.03)	Direct only	(−0.11, −0.3)
Resilience →	Academic Stress Symptoms	−0.03	(−0.07, 0.02)	0.00	(−0.03, 0.03)	−0.03	(−0.07, 0.02)	Full mediation (suppression)	(−0.07, 0.02)

*Bootstrapping sample size = 405. CI, confidence interval*.

Observe in Models 1 and 2 (C, E) that the direct and indirect effects of resilience are greater and positive in nature. In Models 3 and 4 (O, A), effects are positive but smaller. In Model 5 (N), resilience shows robust, negative direct and indirect effects, converting it into a buffering variable (a canceling mediational effect) on stress factors and symptoms.

## Discussion and Conclusion

The proposed hypotheses can reasonably be accepted, based on the results obtained here.

The first *hypothesis*, that the different types of regulatory factors of the BF model would differentially predict the proactive and reactive factors of resilience, was fulfilled overall. These results support prior evidence, which showed that BF personality factors have clear connections to the construct of resilience (77, 78, 118); furthermore, that resilience is positively associated with a well-adjusted personality profile (85, 119). Researchers have shown interest in identifying the individual personality traits or cluster of traits that are positively associated with resilience. These personality traits may be the “antecedents of resilience” and not really components of resilience itself (120). The components of resilience are very necessary in modern life, to navigate through work, study, and relationships in times of uncertainty and lack of predictability (121). Our investigation represents an advance in knowledge, contributing evidence that identifies which components of the BF model have predicted total resilience. The regulatory behavioral components of the BF model (C, E) have predicted proactive factors of resilience (perceived competence, adaptation to change, and perceived control); the non-regulatory BF factors (O, A) were not predictive of the proactive factors of resilience but showed more prediction toward the reactive factors (stress management and spirituality); finally, the BF dysregulatory factor appeared as a significant negative predictor of total resilience and of reactive resilience factors. This is a central contribution toward understanding the relations between the two constructs, and goes in the direction of similar effects that have recently been reported (85, 122, 123). Moreover, indirect support is also found for the regulatory continuum model proposed by *SRL vs. ERL Theory* (65). In short, a well-adjusted personality profile positively predicts resilience.

Regarding the second part of this hypothesis, the same predictive scheme was verified with regard to the factors and symptoms of academic stress. While factors C and A negatively predicted the factors and symptoms of academic stress, factors O and A had a neutral relation to them, and factor N was a negative predictor. The regulatory BF components (C, E) proved to be protective factors against stress (its factors and symptoms), while the non-regulatory factors (O, A) were unrelated to stress factors and symptoms, and the dysregulatory factor (N) appeared as a risk factor for stress (factors and symptoms). These results concur with prior evidence (124, 125). Different studies have found that the C and E personality traits are predictors of academic resilience. Students that exhibit C are well-prepared, they self-regulate, and can maintain calm in the face of stress (119, 126). E and C students have more resources under adverse conditions. The strong association between extraversion and resilience suggests the advantages of positive emotional styles. The capacity for social interaction, close interpersonal relationships, and positive emotions have been found to enable persons to rebound subjectively and physiologically from stressful events (127). Extraversion behavior is positively related to resilience, which in turn facilitates the experience of positive emotions, and encourages seeking out other people and establishing relationships, creating strong social protection networks, which is a critical supportive factor during stressful times. Conscientious students have high self-efficacy and use problem-solving strategies that enhance coping with stressful situations (66, 74). Our results are similar to others because N shows a negative relationship with resilience (19, 84, 85, 128). Consequently, our study contributes toward specifying the non-regulatory and dysregulatory value of the remaining BF components.

The second *hypothesis*, that the proactive and reactive factors of resilience would differentially predict academic stress factors and symptoms, was confirmed. In this case, our evidence represents a valuable contribution in agreement with other previous studies (28, 93), by showing that resilience includes behavioral components that help to endure the negative event (factors that are more reactive in nature) as well as behaviors that help to overcome it (proactive factors, more regulatory in nature). The predictive value of the proactive factors consistently point in this direction, in agreement with prior research (68).

The *third hypothesis*, which stated that the stress factors pertaining to the teaching-learning process would be significant, positive predictors of students' stress symptoms, was also fulfilled. This result is also important, because it confirms that the University teaching-learning context acts as a stress trigger. In line with the previous investigation, stress factors in learning, in their own right, are predictors of students' stress symptoms (129).

The *fourth hypothesis*, regarding the existence of a general structional prediction model, was also acceptably confirmed. Our hypothesis predicted that the personality factors (C, E, O, A, N), in conjunction with resilience, would be diferential predictors of stress symptoms. The negative personality factor (N), jointly with low Resilience, would positively predict stress factors and symptoms, just as this study has confirmed. This general predictive model served to confirm the general relationships between these constructs.

The fifth *hypothesis*, regarding the existence of specific, differential mediational models for each BF factor, was also acceptable. Previous research has already established a clear connection between conscientiousness, extraversion and self-regulation (10, 16, 17). Students may be able to develop their self-regulation skills as a means to better management of their mental health and well-being. This research study has shown a significant mediational effect, the buffering effect of resilience, on the different components of the BF model, in predicting factors and symptoms of University stress. As shown in previous evidence, the greatest predictive power in regard to resilience came from factors C and N, which were positive and negative predictors, respectively (30). In Shi's study, resilience significantly mediated the association of C, A and O, with anxiety symptoms. High levels of A, C and O were associated with high levels of resilience and a lower level of anxiety symptoms. On the other hand, high N, associated with low resilience, correlated to high levels of anxiety. This research, as well as our own, shows that BF factors are not only related directly to states of anxiety and stress, but indirectly through resilience. These results imply that intervention strategies for reducing stress at University should focus on the protective role of certain personality dimensions and in cultivating resilience in students.

### Conclusion

The experience of stress in the University context, due to the difficulty of meeting the demands and requirements of study, is an important phenomenon that has captured the interest of researchers (16). It is important to reduce the impact of stress triggers and encourage students' ability to manage stress. This is essential to their progress, adaptation and success in the University context (130).

The clusters of regulatory BF factors (C, E), non-regulatory factors (O, A) and a dysregulatory factor (N), along with proactive and reactive resilience factors, may act as a *buffer* that helps maintain higher levels of well-being, despite students' elevated levels of perceived stress and impaired mental health functioning (30). Self-regulation has also been linked to good adjustment (e.g., lower psychopathological symptoms) in students of higher education (131).

### Limitations

Several study limitations disadvise broad generalization of these findings. The population in our sample is quite specific and may not be representative of a wider population. Moreover, the sample contained exclusively female students, given that previous research has shown gender differences in these variables (132). By focusing on personality dimensions and their relation to resilience, our study only considered factors and motivations that are internal to the individual. In future studies of resilience, characteristics of the environment and situational factors could be addressed, for example, social support and control over the study environment. The present study, despite certain limitations, contributes to our growing understanding of resilience, as represented in the literature from positive psychology and behavior (19, 133).

Students' stress depends not only on the stressors themselves, but also the synergy between these and students' personal approaches to coping with the situation wherein stress is generated (134). It is important to treat stress at the personal, social and institutional levels.

### Practical Implications for Counseling in the University Context

There are evident implications for educational psychology and counseling in the University setting. Based on the present findings and those of previous research, the BF factors and resilience play an important role in students' levels of stress (10). Certain components of the BF model (C, E) are significantly related to resilience and protect against stress, while others (N) prompt greater vulnerability to stressful situations.

Intervention strategies that focus both on personality traits and on resilience should be implemented in the University context (19). Addressing these variables may be one way to reduce the stress experienced by students –both its prevalence and intensity (81). It is of great importance to detect and address at-risk students—those who have negative affective styles, difficulties in social interaction and who present deficits in self-regulation, self-control, or self-discipline (135, 136).

Education and Health professionals should seek to assess BF factors and resilience, and use intervention programs to further develop resilience in students (19, 66). Guidance services, student mentors, and lecturers can help students to engage in self-awareness about their personality profile and capacity to bounce back (137), in order to strengthen their understanding of their own personal resources for coping with stressful learning environments (138). For instance, they can pursue teaching methods that promote mindfulness (139) and students' cognitive activation [e.g., (140)], or offer other therapeutic or educational approaches. Many universities are already implementing support and intervention in stress management, built around the core concept of resilience (130, 141). The University of Edinburgh (142) offers a Student Resilience model; its declaration of intent states that Resilience “is both a key graduate attribute and an integral part of any transitions framework as it enables students to better cope with the challenges that they will encounter on their unique learning journey.” Likewise, a team from three Australian universities [Curtin University, Queensland University, and University of South Australia; see (143)] have presented a project entitled Building Graduate Resilience for the disrupted future of the twenty-first Century, with the purpose of enhancing resilience in the context of stress in higher education (Project website: www.enhancingresilience.com).

Previous research has indicated that the transition and adjustment to University could be better understood as a trajectory of risk and resilience. The first 2 years can be seen as a challenge, and the final years as an opportunity for growth and recovery (46). Such findings suggest that the first 2 years represent an important transition period where stress management and resilience programs can be highly applicable.

## Data Availability Statement

The raw data supporting the conclusions of this article will be made available by the authors, without undue reservation.

## Ethics Statement

The studies involving human participants were reviewed and approved by http://www.estres.investigacion-psicopedagogica.org/lib/pdf/CERTIFICADO_COMITE_DE_ETICA_UNAV.pdf. The patients/participants provided their written informed consent to participate in this study.

## Author Contributions

JF and JM-V: project managers, design, data analysis, and initial writing. MG-T and RA-G: bibliographic and manuscript review. MV-M and FP-S: data collection. All authors contributed to the article and approved the submitted version.

## Conflict of Interest

The authors declare that the research was conducted in the absence of any commercial or financial relationships that could be construed as a potential conflict of interest.
